# Adaptive Magnetic Resonance-Guided Stereotactic Body Radiotherapy: The Next Step in the Treatment of Renal Cell Carcinoma

**DOI:** 10.3389/fonc.2021.634830

**Published:** 2021-05-11

**Authors:** Brian Keller, Anna M. E. Bruynzeel, Chad Tang, Anand Swaminath, Linda Kerkmeijer, William Chu

**Affiliations:** ^1^ Department of Radiation Oncology, Sunnybrook Health Sciences Centre, University of Toronto, Toronto, ON, Canada; ^2^ Department of Radiation Oncology, Amsterdam University Medical Centers, Amsterdam, Netherlands; ^3^ Department of Radiation Oncology, The University of Texas MD Anderson Cancer Center, Houston, TX, United States; ^4^ Department of Radiation Oncology, Juravinski Cancer Centre, McMaster University, Hamilton, ON, Canada; ^5^ Department of Radiation Oncology, Radboudumc, Nijmegen, Netherlands

**Keywords:** MR-guided radiotherapy, renal cell carcinoma, stereotactic body radiotherapy, MR-linac, image-guided radiotherapy

## Abstract

Adaptive MR-guided radiotherapy (MRgRT) is a new treatment paradigm and its role as a non-invasive treatment option for renal cell carcinoma is evolving. The early clinical experience to date shows that real-time plan adaptation based on the daily MRI anatomy can lead to improved target coverage and normal tissue sparing. Continued technological innovations will further mitigate the challenges of organ motion and enable more advanced treatment adaptation, and potentially lead to enhanced oncologic outcomes and preservation of renal function. Future applications look promising to make a positive clinical impact and further the personalization of radiotherapy in the management of renal cell carcinoma.

## Introduction

Renal cell carcinoma (RCC) is the seventh most common malignancy in the world, where an estimated 400 000 people are diagnosed per year (1). North America has the highest worldwide incidence (age-standardized rate [ASR]: 12 per 100 000) followed by Western Europe (ASR: 9.8) and Australia/New Zealand (ASR: 9.2) (1). The rise in incidence of RCC since the 1980’s has been estimated at approximately 0.5-1% per year, partly attributable to both the increased utilization of cross-sectional imaging leading to incidental findings of small renal masses, and a parallel increase in obesity in Western societies (2, 3). There has also been an increase in the median age of diagnosis (age 65), with the largest increase in patients 70 years or older (4).

Surgical resection remains the gold standard of care in patients with localized RCC. Oftentimes surgery is not possible in an elderly population with other competing medical comorbidities, such as chronic kidney disease (CKD), and carries significant risks of morbidity and/or mortality (5). As a result, options such as active surveillance (AS), or thermal ablation including radiofrequency ablation (RFA) and cryotherapy are considered viable strategies, as demonstrated by their inclusion within the American and European Urological Association guidelines (6, 7). However, tumor size, location and proximity to the renal hilum/vasculature may limit surgical options and percutaneous ablative techniques that require anesthesia. Stereotactic body radiotherapy (SBRT) has emerged as a potential non-invasive option for inoperable patients. A pooled analysis from the International Radiosurgery Oncology Consortium for Kidney (IROCK) has demonstrated SBRT, on conventional conebeam CT (CBCT) linacs or robotic radiosurgery platforms, to be effective in terms of local control (98%), cancer-specific (92%), and progression-free survival (65%) at four years (8). Reported late toxicity (grade ≥3 less than 2%) is minimal, and the impact on renal function (average decrease of 5.5 mL per minute) is comparable to other nonsurgical strategies (9, 10). SBRT has been demonstrated to be effective regardless of tumor size (a limitation of thermal ablative techniques) (11), in patients with solitary kidneys (a limitation for CN or PN) (12), and is well tolerated in an older, medically frail population (13). Prospective trials of RCC SBRT including FASTTRACK II from the Trans-Tasman Radiation Oncology Group (TROG) and the Australian and New Zealand Urogenital and Prostate Cancer Trials Group (ANZUP) (NCT02613819) and RADSTER from Canada (NCT03811665) are ongoing or have completed patient recruitment with results forthcoming in the next few years.

A closer look at the existing pooled analyses suggests there may be further gains to be made. Limitations of these analyses include the absence of pre- and post-treatment comorbidity assessment, retrospective data collection with possible under-reporting of toxicity, and short follow-up. Single fraction SBRT was associated with better progression-free and cancer-specific survival and distant control compared to multi-fraction SBRT, however, patients experienced more nausea. Patients who received a single fraction had better baseline renal function, but demonstrated a trend toward a greater decline compared to patients receiving multiple fractions (8). A further analysis of patients treated with large tumors (>4cm) showed a mean decline in renal function of -7.9 mL per minute; of which a significant proportion of patients had pre-existing stage 3 CKD (11). Could MRgRT permit more utilization of single fraction SBRT (for large tumors in particular) with the potential of further improving oncologic outcomes beyond local control and minimizing the impact on renal function in medically comorbid and inoperable patients?

## Technical Challenges of Irradiating RCC

RCC is traditionally perceived to be radioresistant to conventionally fractionated radiation; however, studies using hypofractionated doses of radiotherapy (RT) demonstrated exponential cell kill (14, 15). Historically large margins were used to ensure that the tumor was irradiated, thus limiting the escalation of dose that could achieve tumor control. This is in part due to large and complex kidney motion (16–19), and highly radiosensitive tissues that surround the kidney and tumor itself, such as the small and large bowel, duodenum and the renal parenchyma. With advances in pretreatment imaging, treatment planning, and implementation of image-guided radiotherapy (IGRT), SBRT was introduced and allowed for delivery of high doses to the tumor. On conventional CBCT-linacs, the internal target volume (ITV) is typically estimated from 4D computed tomography (4DCT) and is the most common passive motion management technique. It represents the treatment volume delineated on all phases of the 4DCT, and is incorporated within the planning target volume (PTV). ITV is based on the assumption that tumor motion estimated during pre-treatment 4DCT acquisition is representative of the motion throughout RT treatment. However, this approach is limited by the inherent low soft-tissue contrast of 4DCT (which may lead to visualization and delineation errors of renal tumors) and on-board CBCT [potentially underestimating intrafraction motion due to respiratory variations (20) and drift (21)], which impacts the reliability of IGRT. As such, larger PTV margins, implanted fiducial markers, or rigid/deformable image registration with multiphasic CT/MRI are options to decrease these uncertainties. Cusumano et al. (22, 23) analyzed the respiratory-induced motion of thoracic and abdominal lesions based on 2D cine-MR (4 images/second) acquired with a 0.35T MR-linac (Viewray, Oakwood Village, OH). In a subset of four kidney patients, the range of 4D-CT motion was 2-9mm craniocaudal (CC) and 1-5mm anteroposterior (AP); the range of MR simulation motion was 5-10mm (CC) and 2-3mm (AP); and 4-9mm (CC) and 2-3mm (AP) during treatment delivery. The data suggests that reliability of the ITV approach may be lower in the abdominal region due to limitations of low soft-tissue contrast and target delineation with 4DCT, and that additional margins of 3mm CC and 2mm AP are required to ensure that renal lesions remain within the ITV for greater than 95% of the time during treatment.

## The Potential of Adaptive MR-guided Radiotherapy for RCC

MR-guided radiotherapy (MRgRT) is a new treatment paradigm that provides high-definition soft-tissue contrast which permits direct visualization of tumors and adjacent radiosensitive organs-at-risk (OAR). MRgRT offers real-time, online monitoring of tumor motion through the different phases of the respiratory cycle and the opportunity for daily adaptation – optimization of tumor targeting and OAR sparing, and dose delivery based on the anatomy from the daily acquired MRI. This may potentially lead to PTV margin reduction and improving the therapeutic ratio. The advantages of online adaptive MRgRT and in which clinical case scenarios maximum benefit will be achieved is yet to be determined.

The MRIdian 0.35T Co-60 MR-linac (Viewray, Oakwood Village, OH) workflow entails patients undergoing both a high-resolution volumetric MR scan and a planning computed tomography (CT) scan with a breath-hold. The CT scan is used for dose calculation purposes and to verify tumor size and shape. The GTV and OARs are delineated on the planning MR image. A PTV is generated by adding a 3 to 5mm margin to the GTV. Daily MRIs are fused with the planning MRI for online adaptation and reoptimization. The system utilizes cine imaging at 4 frames per second in a sagittal plane for real-time anatomic tracking, deformable registration and respiratory-gated, visual patient feedback beam control. The tracking algorithm deforms the anatomical contour on every cine frame and compares it to the gating boundary contour, typically the PTV. Radiation delivery is stopped if the target moves outside the gating boundary, and resumes when it returns to treatment portal (24–27). Early work with lung and abdominal tumors with this system demonstrates at least 95% geometric coverage of GTV (28), and plan adaptation to enhance OAR sparing or to increase PTV coverage on a fraction-by-fraction basis without an increase in acute toxicity (29).

Recently Timmeren et al. (30) retrospectively examined treatment plan quality during the online adaptive re-planning process with a 0.35T Co-60 MR-linac. The MR-guided online adapted plans (n=238) to various targets were compared to the reference plans. The re-optimized plans achieved comparable dosimetric quality to the reference treatment plans, and OAR doses were either comparable or decreased across various tumor sites. The average adaptation time was 24 ± 6 minutes.

Members of the Elekta MR-Linac consortium contribute to the Momentum study (NCT04075305) (31) which is a prospective registry to capture all patient-related data as well as technical data to facilitate the development and implementation of MRgRT. Some patient selection and workflow criteria have been outlined by Hall et al. (32) in their treatment of liver and pancreas cancers using the Unity 1.5T system (Elekta, Stockholm, Sweden). A patient may be a potential candidate for MR-Linac radiotherapy if their lesion is difficult or impossible to visualize on a non-contrast CT, the lesion is in close proximity (within 1 cm) of a radiosensitive normal structure, and the patient is amenable to clinical trial participation. The 1.5T MR-linac provides two workflow solutions, namely, the adapt-to-position (ATP) or adapt-to-shape (ATS) workflows as previously described (33). The ATP workflow is a dose re-calculation after an image fusion based on the daily MRI, but it does not involve re-contouring on the daily MRI. It is ideal for those scenarios where there is minimal inter-fractional variation, a low chance of size variations and a reasonable distance between a mobile OAR and the target. The ATS workflow involves re-contouring and re-optimization of a new treatment plan based on the daily MRI. The ATS workflow may be ideal in a scenario where inter-fractional variations could be significant, such as a rapidly changing tumor size or close proximity to air cavities or mobile gastrointestinal structures.

Hall et al. (32) recently reported on ten patients (13 targets) treated with MRgRT for primary and secondary tumors of the liver and pancreas with a 1.5T MR-linac. Patients underwent 4DCT and MRI simulation, and an ITV method for motion management was used based on the 4DCT image dataset. PTV margins ranged from 3 to 5 mm. Daily adaptation was accomplished with the acquisition of pretreatment 4D MRIs, where motion-averaged or mid-position images were reconstructed and used for plan optimization, with either an ATP or ATS approach. The decision to use ATS was based on tumor proximity (3-5mm) to a mobile OAR (luminal GI structure) with variable daily position, proximity to an air cavity, and variable tumor size and position. Real-time monitoring of the target during treatment was done with cine MRIs in three perpendicular planes through the centre of the PTV. The median treatment time for the ATS workflow was 64 minutes. Currently, only free-breathing methods of motion management (with or without abdominal compression) are clinically feasible on the 1.5T MR-linac, while gating solutions are in preparation.

It is a natural evolution to apply MRgRT for kidney tumors alongside other abdominal/pelvic targets that share the same adjacent radiosensitive OARs (duodenum, small bowel, large bowel). The high-definition soft-tissue contrast of MRgRT permits better visualization of kidney substructures — such as the renal hilum (vasculature and collecting system) and parenchyma — that are hard to differentiate with conventional cone beam CT-guided radiotherapy, which may lead to increased tissue sparing and preservation of renal function.

## Early Clinical Experience With MR-Guided Radiotherapy for RCC

Rudra et al. (34) published the first case report of a RCC patient that was treated with a 0.35T Co-60 MR-linac utilizing an end-expiration gating technique to deliver a dose of 40 Gy in 5 fractions. The treatment target was the GTV surrounded by a 5 mm gating boundary. The larger gating boundary resulted in less beam-on interruption and shorter treatment times, at the expense of irradiating more normal tissue. Typical gating margins ranged from 3 to 5 mm. For treatment planning 4D CT and MRI data sets were fused for contouring and dose calculation. The patient had lung and brain metastases, declined cytoreductive nephrectomy and continuation of nivolumab, and was treated with SBRT for the purpose of cytoreduction. No acute or late toxicities were reported, and the tumor and renal function remained stable 6 months after SBRT.

Tetar et al. (35) are the first group to report the outcomes of 36 primary RCC patients treated to a dose of 40Gy in 5 fractions on a 0.35T Co-60 MR-linac. The mean age of the cohort was 78.1 years and tumor diameter was 5.6cm (T1a: 5 patients; T1b: 23 patients; T2a: 8 patients). With a median follow-up of 16.4 months, the 1-year local control was 95.2%, freedom from progression was 91% and overall survival was 91.2%. One patient experienced acute grade ≥2 nausea, and no other acute or late toxicities were reported. Baseline mean eGFR was 55.3 mL/min/1.73 m^2^ (SD ±19.0), and the mean decline in eGFR post-MRgRT was 6.0 mL/min/1.73 m^2^. While the follow-up interval is short, oncologic outcomes and preservation of renal function in this cohort of mainly large tumors are favorable and consistent with a recent analysis of RCC SBRT (8, 11).

**Figure 1:**
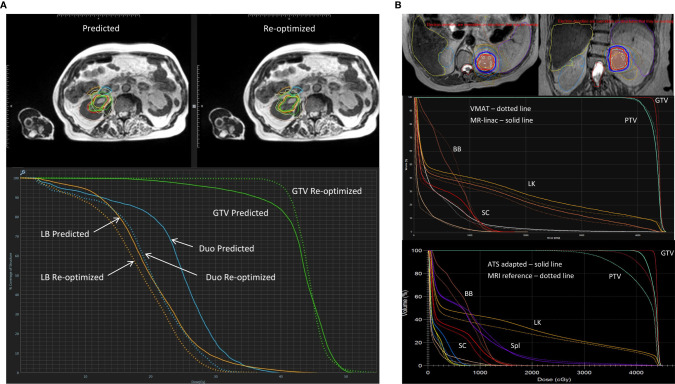
Representative MRgRT treatment plans for RCC patients with **(A)** 40 Gy in 5 fractions on a 0.35T MR-linac (MRIdian, ViewRay, Oakwood Village, OH) and **(B)** 42 Gy in 3 fractions on a 1.5T MR-linac (Unity, Elekta, Stockholm, Sweden). **(A)** Top panel showing axial MRIs of a predicted and re-optimized plan of a right-sided RCC: GTV (green contour), duodenum (blue contour) and large bowel (orange contour); Bottom panel showing the corresponding DVH of the predicted (solid line) and re-optimized (dotted line) plans with improved GTV (green) coverage, and sparing of the duodenum (Duo - blue) and large bowel (LB - orange). *Reproduced with permission from AME Bruynzeel (Amersterdam UMC).* **(B)** Axial (left) and coronal (right) MRI treatment plan of a left-sided RCC showing ITV (brown color wash) and PTV (light green color wash) with isodose lines: 42Gy (red) and 36Gy (blue); Middle panel showing a DVH of VMAT (dotted line) and 1.5T MR-linac treatment plans (solid line); Bottom panel showing a DVH of an ATS adaptive (solid line) and reference plan (dotted line) with improved ITV (dark red) and PTV (light green) coverage, and equivalent sparing of the left kidney (LK – orange), spleen (Spl – purple), bowel bag (BB – brown) and spinal cord (SC - bright red). *Reproduced with permission from C Tang (MD Anderson Cancer Center)* (31).

Prior to the delivery of each treatment fraction patients completed a short breath-hold MR scan, rigid registration was performed on the GTV and the OAR contours were propagated to the daily MRI scan using deformable registration. Routine plan re-optimization was undertaken where the treating radiation oncologist adjusted the GTV and OAR contours within 2 cm of the PTV. A baseline IMRT plan was recalculated on the new anatomy from the daily MRI (predicted plan), and then re-optimized using the target and OAR optimization objectives of the baseline plan (re-optimized plan). The priority of plan re-optimization was to minimize high dose to OARs, even at the expense of decreased PTV coverage. The re-optimized plan was used for treatment delivery. MRgRT was delivered with respiratory gating where the gated structure was either the kidney itself, or the primary tumor if visible. Gating was augmented by visual and/or auditory feedback where patients were able to visualize the gated structure and the gating boundary, generally corresponding to the PTV (3mm). Treatment times for these patients ranged from 30–45 minutes for real time contour propagation, plan re-optimization and treatment delivery. **Figure 1A** shows a predicted and re-optimized plan and DVH for one treatment fraction of a right-sided RCC that highlights the improvement in GTV coverage and sparing of the duodenum and large bowel with plan adaptation.

The University of Texas MD Anderson Cancer Center is building experience in the treatment of primary kidney tumors and metastatic lesions within the kidney parenchyma on a 1.5T MR-linac. In collaboration with the urology department, non-operable RCC patients are currently being enrolled into the MRI-MARK trial evaluating the feasibility and effectiveness of MRI-based SBRT at a dose of 42 Gy in 3 fractions to the gross tumor volume (NCT04580836). An ITV method for motion management is employed and daily adaptation is done with the acquisition of pretreatment free-breathing T2 MRIs, followed by an adapt-to-position (ATP) or adapt-to-shape (ATS) workflow. Monitoring is achieved using real-time cine MRI with 3 orthogonal planes through the PTV during beam-on. **Figure 1B** shows the dose distribution for a left mid-upper pole RCC, and a DVH demonstrating the ability to achieve equivalent target coverage and OAR sparing with a MR-linac and standard VMAT treatment plan. With ATS plan adaption, GTV and PTV coverage can be improved while maintaining OAR sparing.

## Future Opportunities

Therapies maximizing nephron-sparing is a priority for RCC patients in whom the prevalence of CKD is high (36). More efficacious and safer SBRT can be achieved with dose escalation and a reduction in margins, and requires MRgRT systems to advance with enhanced MRI sequences, intrafraction tracking and gating, and treatment adaptation. Developmental work in these areas is ongoing.

**Figure 2:**
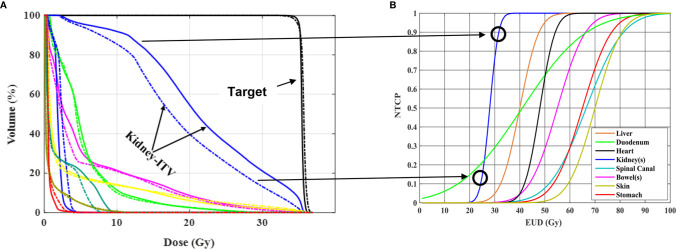
Radiobiological impact of RCC motion tracking in **(A)** and patient treatment in **(B)** both utilizing a 1.5 T MR-linac Unity system (Elekta, Stockholm, Sweden). **(A)** *Adapted with permission from Al-Ward et al.* (37). On the left are shown DVHs resulting from two different treatment planning methodologies, one accounting for the ITV method of motion management (solid curve) and the other accounting for tumor tracking (dashed curve) for one of three kidney patients investigated. The sparing in irradiated normal kidney by using tracking results in a reduction in predicted normal tissue complication probability as shown on the right. Such reduction can be viewed as a way to reduce normal kidney toxicity or a way to maintain current toxicity but increase the dose delivered. This is simulated data representative of an ideal case scenario, indicating the potential benefits that could be achieved using an MR-guided motion tracking delivery strategy.

Al-Ward et al. (37) evaluated and quantified the potential radiobiological advantages of tumor tracking using the 1.5T MR-linac (Unity, Elekta, Stockholm, Sweden) for abdominal tumors (3 liver, 3 pancreas, 3 kidney). The investigators applied two planning methods, the conventional ITV method and a simulated tracking method (STT). The STT method was developed initially for lung tumor tracking in an MR-Linac and accounts for 8 phases of the breathing cycle, where more weight is applied to those phases where more time is spent. Similar methodology was then applied to the abdominal/pelvic targets. The average reduction in normal volume irradiated for kidney tumor patients due to tracking was 26.9%. The authors report that a normal tissue complication probability (NTCP) benefit due to tracking, was observed in 26% of the data. For all three disease sites, the maximum NTCP improvements were for the normal kidney, the bowels and the duodenum, with reductions in associated toxicities of 79% (radiation nephropathy) (38, 39), 69% (stricture/fistula) (38, 40) and 25% (ulceration) (38, 41), respectively. Even though this was a simulation study using a well-validated planning system, it indicates the potential benefits, in a best case scenario, that may be achieved in the reduction of side effects and/or an increase in tumor control probability if real-time tumor tracking is implemented (**Figure 2**).

Prins et al. (21) evaluated two motion management techniques, tumor trailing and respiratory tracking, in 15 RCC patients simulated for single-fraction, MRI-based SBRT within a 25-minute treatment time with free breathing. The largest respiratory tumor motion was observed along the CC direction with a median 95% maximum amplitude of approximately 12mm. Without mechanical immobilization, intrafraction drift accounted for 75% of the total intrafraction motion margin for online mid-position-based SBRT treatments. The described study, and a previous dose accumulation study highlight the importance of accounting for intrafraction motion and its impact on dose accumulation. These studies strengthen the case for online motion monitoring and real-time plan adaptation (21, 42). In a free-breathing treatment scenario the margin calculations show that a 6.1mm PTV margin would be required to account for the systematic and random errors of drift and respiratory motion, and could be reduced to 1.5mm with tumor trailing.

Further technical development will enable the opportunity to increase the utilization of single fraction SBRT for RCC (small and large) and enable future comparative studies to thermal ablative procedures. The next step in the evolution of MRgRT for RCC will be the ability to treat: multiple targets in the ipsilateral and/or contralateral kidney; oligometastatic (43) or oligoprogressive metastases{Palma, 2020 #677;Cheung, 2020 #687} (44) simultaneously; and large primary lesions in metastatic RCC (mRCC) that are not amenable for upfront cytoreductive nephrectomy (CN). With respect to the last scenario, results from the SURTIME (45) and CARMENA (46) trials, have led to a decrease in CN for International Metastatic RCC Database Consortium (IMDC) intermediate- and poor-risk patients. Based on the results of the Checkmate-214 trial (47), first-line treatment of mRCC is now combination immunotherapy with ipilimumab and nivolumab in intermediate/poor risk patients compared to sunitinib previously. “Cytoreductive’ SBRT to the primary kidney lesion may be a novel treatment strategy to induce an enhanced and synergistic systemic anti-tumor immune response (an abscopal effect). This has been observed in patients with melanoma receiving anti–CTLA-4 therapy (48) as well as patients with RCC (49, 50). Putative mechanisms for this response include immune stimulation by novel neoantigens or pre-existing antigen-presenting cells, upregulation of calreticulin and CD8^+^ proliferating T cells and other key immune-modulating cytokines (51, 52). The combination of nivolumab/ipilimumab along with cytoreductive SBRT to the primary lesion for mRCC is currently being evaluated in a randomized, phase II clinical trial (CYTOSHRINK NCT04090710) (53). MRgRT within this treatment paradigm may improve the therapeutic ratio by maximizing tumor coverage (generally large or unresectable lesions) while minimizing dose to OARs and the risk of combined radiation-immunotherapy treatment-related toxicities (for example, acute kidney injury and progression of CKD). Functional MRI for the diagnosis and prediction of treatment response for RCC are areas of ongoing investigation (54). Acquiring functional imaging studies on a 0.35T and 1.5T MR-linac during treatment is feasible (32, 55, 56). With consensus guidelines for image acquisition and quantification (57), MRgRT offers a unique opportunity to assess novel imaging biomarkers of response and toxicity in conjunction with serological correlates during SBRT alone or in combination with immunotherapy.

## Summary

The role of MRgRT in the treatment of RCC continues to evolve. MRgRT can potentially facilitate dose escalation and smaller treatment margins by overcoming the challenge of complex kidney motion, and reduce treatment-related toxicities by carefully evaluating and sparing critical OARs in real time. In the primary setting, this technology will help advance the use of SBRT for small and large renal tumors with potentially less renal toxicity, and improve the therapeutic ratio which will facilitate future comparative effectiveness studies versus other ablative modalities. In the metastatic setting, the benefits of MRgRT for oligometastatic or oligoprogressive tumors, and in combination with immunotherapy, may even be more pronounced where online tumor monitoring and daily adaptation to optimize dose delivery and OAR sparing may further mitigate toxicity. Such an approach would allow for potentially more effective combined modality therapy and brings us closer to realizing the promise of high-precision and personalized medicine in the field of radiation oncology.

## Data Availability Statement

The original contributions presented in the study are included in the article/supplementary material. Further inquiries can be directed to the corresponding author.

## Ethics Statement

Ethical approval was not provided for this study on human participants because this perspective highlights previously published and ongoing studies that have received local ethics approval. Written informed consent for participation was not required for this study in accordance with the national legislation and the institutional requirements.

## Author Contributions

All authors contributed to the article and approved the submitted version.

## Conflict of Interest

AB reports personal fees from ViewRay Inc., outside the submitted work. AS has received honoraria from Bristol Myers Squibb, AstraZeneca, and Eisai, and trial funding in kind from Bristol Myers Squibb. BK, CT, LK and WC are members of the Elekta MR-Linac Consortium, and Elekta financially supports projects in the member institutes. BK and WC have received travel support from Elekta.

## References

[1] Padala SA Barsouk A Thandra KC Saginala K Mohammed A Vakiti A. Epidemiology of Renal Cell Carcinoma. World J Oncol (2020) 11(3):79–87. 10.14740/wjon1279 32494314 PMC7239575

[2] Hollingsworth JM Miller DC Daignault S Hollenbeck BK. Rising incidence of small renal masses: a need to reassess treatment effect. J Natl Cancer Inst (2006) 98(18):1331–4. 10.1093/jnci/djj362 16985252

[3] Klinghoffer Z Yang B Kapoor A Pinthus JH. Obesity and renal cell carcinoma: epidemiology, underlying mechanisms and management considerations. Expert Rev Anticancer Ther (2009) 9(7):975–87. 10.1586/era.09.51 19589036

[4] Capitanio U Bensalah K Bex A Boorjian SA Bray F Coleman J. Epidemiology of Renal Cell Carcinoma. Eur Urol (2019) 75(1):74–84. 10.1016/j.eururo.2018.08.036 30243799 PMC8397918

[5] Rendon RA Kapoor A Breau R Leveridge M Feifer A Black PC. Surgical management of renal cell carcinoma: Canadian Kidney Cancer Forum Consensus. Can Urol Assoc J (2014) 8(5-6):E398–412. 10.5489/cuaj.1894 PMC4081255 25024794

[6] Campbell S Uzzo RG Allaf ME Bass EB Cadeddu JA Chang A. Renal Mass and Localized Renal Cancer: AUA Guideline. J Urol (2017) 198(3):520–9. 10.1016/j.juro.2017.04.100 28479239

[7] Ljungberg B Albiges L Abu-Ghanem Y Bensalah K Dabestani S Fernandez-Pello S. European Association of Urology Guidelines on Renal Cell Carcinoma: The 2019 Update. Eur Urol (2019) 75(5):799–810. 10.1016/j.eururo.2019.02.011 30803729

[8] Siva S Louie AV Waner A Muacevic A Gandhidasan S Ponsky L. Pooled analysis of stereotactic ablative radiotherapy for primary renal cell carcinoma: A report from the International Radiosurgery Oncology Consortium for Kidney (IROCK). Cancer (2018) 124(5):934–42. 10.1002/cncr.31156 29266183

[9] Chang JH Cheung P Erler D Sonier M Korol R Chu W. Stereotactic Ablative Body Radiotherapy for Primary Renal Cell Carcinoma in Non-surgical Candidates: Initial Clinical Experience. Clin Oncol (R Coll Radiol) (2016) 28(9):e109–14. 10.1016/j.clon.2016.04.002 27131756

[10] Ponsky L Lo SS Zhang Y Schluchter M Liu Y Patel R. Phase I dose-escalation study of stereotactic body radiotherapy (SBRT) for poor surgical candidates with localized renal cell carcinoma. Radiother Oncol (2015) 117(1):183–7. 10.1016/j.radonc.2015.08.030 26362723

[11] Siva S Correa RJM Warner A Staehler M Ellis RJ Ponsky L. Stereotactic Ablative Radiotherapy for ‡T1b Primary Renal Cell Carcinoma: A Report From the International Radiosurgery Oncology Consortium for Kidney (IROCK). Int J Radiat Oncol Biol Phys (2020) 108(4):947–9. 10.1016/j.ijrobp.2020.06.014 32562838

[12] Correa RJM Louie AV Staehler M Warner A Gandhidasan S Ponsky L. Stereotactic Radiotherapy as a Treatment Option for Renal Tumors in the Solitary Kidney: A Multicenter Analysis from the IROCK. J Urol (2019) 201(6):1097–104. 10.1097/JU.0000000000000111 30741849

[13] Swaminath A Niglas M Cheung P Erler D Korol R Blain J. Patient-Reported Quality of Life Following Stereotactic Body Radiation Therapy for Primary Kidney Cancer: Results from a Prospective Cohort Study. Int J Radiat Oncol Biol Physics (2018) 102(3, Supplement):e93–e4. 10.1016/j.ijrobp.2018.07.366 33775496

[14] Ning S Trisler K Wessels B Knox S. Radiobiologic studies of radioimmunotherapy and external beam radiotherapy in vitro and in vivo in human renal cell carcinoma xenografts. Cancer (1997) 80:2519–28. 10.1002/(SICI)1097-0142(19971215)80:12+<2519::AID-CNCR26>3.0.CO;2-E 9406705

[15] Walsh L Stanfield JL Cho LC Chang CH Forster K Kabbani W. Efficacy of ablative high-dose-per-fraction radiation for implanted human renal cell cancer in a nude mouse model. Eur Urol (2006) 50(4):795–800; discussion. 10.1016/j.eururo.2006.03.021 16632182

[16] Sonier M Chu W Lalani N Erler D Cheung P Korol R. Evaluation of kidney motion and target localization in abdominal SBRT patients. J Appl Clin Med Physics (2016) 17(6):429–33. 10.1120/jacmp.v17i6.6406 PMC5690515 27929514

[17] Siva S Pham D Gill S Bressel M Dang K Devereux T. An analysis of respiratory induced kidney motion on four-dimensional computed tomography and its implications for stereotactic kidney radiotherapy. Radiat Oncol (London England) (2013) 8:248. 10.1186/1748-717X-8-248 PMC3829388 24160868

[18] Pham D Kron T Styles C Whitaker M Bressel M Foroudi F. The use of dual vacuum stabilization device to reduce kidney motion for stereotactic radiotherapy planning. Technol Cancer Res Treat (2015) 14(2):149–57. 10.7785/tcrt.2012.500410 24502551

[19] Pham D Kron T Bressel M Foroudi F Hardcastle N Schneider M. Image guidance and stabilization for stereotactic ablative body radiation therapy (SABR) treatment of primary kidney cancer. Pract Radiat Oncol (2015) 5(6):e597–605. 10.1016/j.prro.2015.08.002 26547828

[20] Sonier M Chu W Lalani N Erler D Cheung P Korol R. Implementation of a volumetric modulated arc therapy treatment planning solution for kidney and adrenal stereotactic body radiation therapy. Med Dosim (2016) 41(4):323–8. 10.1016/j.meddos.2016.09.001 27745995

[21] Prins FM Stemkens B Kerkmeijer LGW Barendrecht MM de Boer HJ Vonken EPA. Intrafraction Motion Management of Renal Cell Carcinoma With Magnetic Resonance Imaging-Guided Stereotactic Body Radiation Therapy. Pract Radiat Oncol (2019) 9(1):e55–61. 10.1016/j.prro.2018.09.002 30261329

[22] Cusumano D Dhont J Boldrini L Chilorio G Romano A Votta C. Reliability of ITV approach to varying treatment fraction time: a retrospective analysis based on 2D cine MR images. Radiat Oncol (2020) 15:152. 10.1186/s13014-020-01530-6 32532334 PMC7291491

[23] Cusumano D Dhont J Boldrini L Chiloiro G Teodoli S Massaccesi M. Predicting tumour motion during the whole radiotherapy treatment: a systematic approach for thoracic and abdominal lesions based on real time MR. Radiother Oncol (2018) 129(3):456–62. 10.1016/j.radonc.2018.07.025 30144955

[24] Olsen J Green O Kashani R. World’s first application of MR-Guidance for Radiotherapy. Missouri Med (2015) 112(5):358–60. PMC6167237 26606816

[25] van Sornsen de Koste JR Palacios MA Bruynzeel AME Slotman BJ Senan S. Lagerwaard FJ MR-guided Gated Stereotactic Radiation Therapy Delivery for Lung, Adrenal, and Pancreatic Tumors: A Geometric Analysis. Radiat Oncol Int J Biol Phys (2018) 102(4):858–66. 10.1016/j.ijrobp.2018.05.048 30061007

[26] Green OL Rankine LJ Cai B Curcuru A Kashani R Rodriguez V. First clinical implementation of real-time, real anatomy tracking and radiation beam control. Med Phys (2018) 45(8):3728–40. 10.1002/mp.13002 29807390

[27] Mutic S Dempsey JF. The ViewRay system: magnetic resonance-guided and controlled radiotherapy. Semin Radiat Oncol (2014) 24(3):196–9. 10.1016/j.semradonc.2014.02.008 24931092

[28] Kost S Dörr W Keinert K Glaser FH Endert G Herrmanns T. Effect of dose and dose distribution in damage to the kidney following abdominal radiotherapy. Int J Radiat Biol (2002) 78(8):695–702. 10.1080/09553000210134791 12194753

[29] Henke L Kashani R Robinson C Curcuru A DeWees T Bradley J. Phase I trial of stereotactic MR-guided online adaptive radiation therapy (SMART) for the treatment of oligometastatic or unresectable primary malignancies of the abdomen. Radiother Oncol (2017) 126(3):519–26. 10.1016/j.radonc.2017.11.032 29277446

[30] van Timmeren JE Chamberlain M Krayenbuehl J Wilke L Ehrbar S Bogowicz M. Treatment plan quality during online adaptive re-planning. Radiat Oncol (London England) (2020) 15:203. 10.1186/s13014-020-01641-0 PMC7441614 32825848

[31] de Mol van Otterloo S Christodouleas J Blezer ELA Akhiat H Brown K Choudhury A. The MOMENTUM Study: An International Registry for the Evidence-Based Introduction of MR-Guided Adaptive Therapy. Front Oncol (2020) 10:1328. 10.3389/fonc.2020.01328 33014774 PMC7505056

[32] Hall WA Straza MW Chen X Mickervicius N Erickson B Schults C. Initial clinical experience of Stereotactic Body Radiation Therapy (SBRT) for liver metastases, primary liver malignancy, and pancreatic cancer with 4D-MRI based online adaptation and real-time MRI monitoring using a 1.5 Tesla MR-Linac. PLOS ONE (2020) 15(8):e0236570. 10.1371/journal.pone.0236570 32764748 PMC7413561

[33] Winkel D Bol GH Kroon PS van Asselen B Hackett SS Werensteijn-Honingh AM. Adaptive radiotherapy: The Elekta Unity MR-linac concept. Clin Transl Radiat Oncol (2019) 18:54–9. 10.1016/j.ctro.2019.04.001 PMC6630157 31341976

[34] Rudra S Fischer-Valuck B Pachynski R Daly M Green O. Magnetic Resonance Image Guided Stereotactic Body Radiation Therapy to the Primary Renal Mass in Metastatic Renal Cell Carcinoma. Adv Radiat Oncol (2019) 4(4):566–70. 10.1016/j.adro.2019.04.002 PMC6817517 31673649

[35] Tetar SU Bohoudi O Senan S Palacios MA Oei SS van der Wel A. The Role of Daily Adaptive Stereotactic MR-Guided Radiotherapy for Renal Cell Cancer. Cancers (2020) 12:2763. 10.3390/cancers12102763 PMC7601380 32992844

[36] Porta C Bamias A Danesh FR Debska-Slizien A Gallieni M Gertz MA. KDIGO Controversies Conference on onco-nephrology: understanding kidney impairment and solid-organ malignancies, and managing kidney cancer. Kidney Int (2020) 98:1108–19. 10.1016/j.kint.2020.06.046 33126977

[37] Al-Ward S Wronski M Ahmad SB Myrehaug S Chu W Sahgal A. The radiobiological impact of motion tracking of liver, pancreas and kidney SBRT tumors in a MR-linac. Phys Med Biol (2018) 63(215022):11. 10.1088/1361-6560/aae7fd 30375365

[38] Burman C Kutcher GJ Emami B Goitein M. Fitting of normal tissue tolerance data to an analytic function. Int J Radiat Oncol • Biol • Physics (1991) 21(1):123–35. 10.1016/0360-3016(91)90172-Z 2032883

[39] Cohen EP Robbins MEC. Radiation nephropathy. Semin Nephrol (2003) 23(5):486–99. 10.1016/S0270-9295(03)00093-7 13680538

[40] Kuku S Fragkos C McCormack M Forbes A. Radiation-induced bowel injury: the impact of radiotherapy on survivorship after treatment for gynaecological cancers. Br J Cancer (2013) 109(6):1504–12. 10.1038/bjc.2013.491 PMC3777000 24002603

[41] Kavanagh BD Pan CC Dawson LA Das SK Li XA Ten Haken RK. Radiation dose-volume effects in the stomach and small bowel. Int J Radiat Oncol • Biol • Physics (2010) 76(3 Suppl):S101–7. 10.1016/j.ijrobp.2009.05.071 20171503

[42] Stemkens B Glitzner M Kontaxis C de Senneville BD Prins FM Crijns SPM. Effect of intra-fraction motion on the accumulated dose for free-breathing MR-guided stereotactic body radiation therapy of renal-cell carcinoma. Phys Med Biol (2017) 62(18):7407–24. 10.1088/1361-6560/aa83f7 28771144

[43] Palma DA Olson R Harrow S Gaede S Louie AV Haasbeek C. Stereotactic Ablative Radiotherapy for the Comprehensive Treatment of Oligometastatic Cancers: Long-Term Results of the SABR-COMET Phase II Randomized Trial. J Clin Oncol Off J Am Soc Clin Oncol (2020) 38(25):2830–8. 10.1101/2020.03.26.20044305 PMC7460150 32484754

[44] Cheung P Patel S North SA Sahgal A Chu W Soliman H. A phase II multicenter study of stereotactic radiotherapy (SRT) for oligoprogression in metastatic renal cell cancer (mRCC) patients receiving tyrosine kinase inhibitor (TKI) therapy. J Clin Oncol (2020) 38(15_suppl):5065. 10.1200/JCO.2020.38.15_suppl.5065 34399998

[45] Bex A Mulders P Jewett M Wagstaff J van Thienen JV Blank CU. Comparison of Immediate vs Deferred Cytoreductive Nephrectomy in Patients With Synchronous Metastatic Renal Cell Carcinoma Receiving Sunitinib: The SURTIME Randomized Clinical Trial. JAMA Oncol (2019) 5(2):164–70. 10.1001/jamaoncol.2018.5543 PMC6439568 30543350

[46] Mejean A Ravaud A Thezenas S Colas S Beauval JB Bensalah K. Sunitinib Alone or after Nephrectomy in Metastatic Renal-Cell Carcinoma. N Engl J Med (2018) 379(5):417–27. 10.1056/NEJMoa1803675 29860937

[47] Motzer RJ Tannir N McDermott DF Frontera OA Melichar B Choueiri TK. CheckMate 214 Investigators. Nivolumab plus Ipilimumab versus Sunitinib in Advanced Renal-Cell Carcinoma. N Engl J Med (2018) 378(14):1277–90. 10.1056/NEJMoa1712126 PMC5972549 29562145

[48] Koller KM Mackley HB Liu J Wagner H Talamo G Schell TD. Improved survival and complete response rates in patients with advanced melanoma treated with concurrent ipilimumab and radiotherapy versus ipilimumab alone. Cancer Biol Ther (2017) 18(1):36–42. 10.1080/15384047.2016.1264543 27905824 PMC5323007

[49] Xie G Gu D Zhang L Chen S Wu D. A rapid and systemic complete response to stereotactic body radiation therapy and pembrolizumab in a patient with metastatic renal cell carcinoma. Cancer Biol Ther (2017) 18(8):547–51. 10.1080/15384047.2017.1345389 PMC5652971 28665741

[50] Hammers HJ Vonmerveldt D Ahn C Nadal RM Drake CG Folkert MR. Combination of dual immune checkpoint inhibition (ICI) with stereotactic radiation (SBRT) in metastatic renal cell carcinoma (mRCC) (RADVAX RCC). J Clin Oncol (2020) 38(6_suppl):614. 10.1200/JCO.2020.38.6_suppl.614

[51] Singh AK Winslow TB Kermany MH Goritz V Heit L Miller A. A Pilot Study of Stereotactic Body Radiation Therapy Combined with Cytoreductive Nephrectomy for Metastatic Renal Cell Carcinoma. Clin Cancer Res (2017) 23(17):5055–65. 10.1158/1078-0432.CCR-16-2946 PMC5581708 28630212

[52] Tang C Welsh JW de Groot P Massarelli E Chang JY Hess KR. Ipilimumab with Stereotactic Ablative Radiation Therapy: Phase I Results and Immunologic Correlates from Peripheral T Cells. Clin Cancer Res (2017) 23(6):1388–96. 10.1158/1078-0432.CCR-16-1432 PMC5355002 27649551

[53] Lalani AKA Swaminath A Pond GR Kapoor A Chu W Bramson JL. Phase II trial of cytoreductive stereotactic hypofractionated radiotherapy with combination ipilimumab and nivolumab for metastatic kidney cancer (CYTOSHRINK). J Clin Oncol (2020) 38(6 Suppl):TPS761–TPS. 10.1200/JCO.2020.38.6_suppl.TPS761

[54] Wu Y Kwon YS Labib M Foran DJ Singer EA. Magnetic Resonance Imaging as a Biomarker for Renal Cell Carcinoma. Dis Markers (2015) 2015:648495. 10.1155/2015/648495 26609190 PMC4644550

[55] Yang Y Cao M Sheng K Gao Y Chen A Kamrava M. Longitudinal diffusion MRI for treatment response assessment: Preliminary experience using an MRI-guided tri-cobalt 60 radiotherapy system. Med Phys (2016) 43(3):1369–73. 10.1118/1.4942381 PMC6961701 26936721

[56] Kooreman ES van Houdt PJ Nowee ME van Pelt VWJ Tijssen RHN Paulson ES. Feasibility and accuracy of quantitative imaging on a 1.5 T MR-linear accelerator. Radiother Oncol (2019) 133:156–62. 10.1016/j.radonc.2019.01.011 30935572

[57] Kooreman ES van Houdt PJ Keesman R Pos FJ van pelt VWJ Nowee ME. ADC measurements on the Unity MR-linac – A recommendation on behalf of the Elekta Unity MR-linac consortium. Radiother Oncol (2020) 153:106–13. 10.1016/j.radonc.2020.09.046 PMC8327388 33017604

